# SNP-based analysis of genetic diversity reveals important alleles associated with seed size in rice

**DOI:** 10.1186/s12870-016-0779-3

**Published:** 2016-04-19

**Authors:** Weijie Tang, Tingting Wu, Jian Ye, Juan Sun, Yue Jiang, Jun Yu, Jianpeng Tang, Gaoming Chen, Chunming Wang, Jianmin Wan

**Affiliations:** State Key Laboratory of Crop Genetics and Germplasm Enhancement, Nanjing Agricultural University, 210095 Nanjing, China; State Key Laboratory of Plant Genomics, Institute of Microbiology, Chinese Academy of Sciences, 100101 Beijing, China; Jiangsu Collaborative Innovation Center for Modern Crop Production, Nanjing, China; National Key Facility for Crop Gene Resources and Genetic Improvement, Institute of Crop Science, Chinese Academy of Agricultural Sciences, Beijing, 100081 China

**Keywords:** SNP, miRNA, Genetic diversity, Seed size, Rice

## Abstract

**Background:**

Single-nucleotide polymorphisms (SNPs) have become the genetic markers of choice in various genetic, ecological, and evolutionary studies. Genotyping-by-sequencing (GBS) is a next-generation-sequencing based method that takes advantage of reduced representation to enable high-throughput genotyping using a large number of SNP markers.

**Results:**

In the present study, the distribution of non-redundant SNPs in the parents of 12 rice recombination line populations was evaluated through GBS. A total of 45 Gigabites of nucleotide sequences conservatively provided satisfactory genotyping of rice SNPs. By assembling to the genomes of reference genomes of *japonica* Nipponbare, we detected 22,682 polymorphic SNPs that may be utilized for QTL/gene mapping with the Recombinant Inbred Lines (RIL) populations derived from these parental lines. Meanwhile, we identified polymorphic SNPs with large effects on protein-coding and miRNA genes. To validate the effect of the polymorphic SNPs, we further investigated a SNP (chr4:28,894,757) at the miRNA binding site in the 3′-UTR region of the locus *Os4g48460*, which is associated with rice seed size. *Os4g48460* encodes a putative cytochrome P450, *CYP704A3*. Direct degradation of the 3′-UTR of the *CYP704A3* gene by a miRNA (*osa-miRf10422-akr*) was validated by *in planta* mRNA degradation assay. We also showed that rice seeds of longer lengths may be produced by downregulating *CYP704A3* via RNAi.

**Conclusions:**

Our study has identified the genome-wide SNPs by GBS of the parental varieties of RIL populations and identified *CYP704A3*, a miRNA-regulated gene that is responsible for rice seed length.

**Electronic supplementary material:**

The online version of this article (doi:10.1186/s12870-016-0779-3) contains supplementary material, which is available to authorized users.

## Background

Rice is the first crop plant from which a high-quality reference genome sequence from a single variety has been produced. Single nucleotide polymorphisms (SNPs) may be functionally responsible for specific traits or phenotypes, or they may be informative in tracing the evolutionary history of a species or the pedigree of a variety. SNPs are rapidly replacing simple sequence repeats (SSRs) because these are more abundant, stable, amenable to automation, efficient, and increasingly cost-effective [1].

SNPs have become the genetic marker of choice in the analysis of partially or completely sequenced genomes due to its ubiquity in the genome [2]. In rice, the primary sequencing data that led to the first whole-genome SNP discovery was derived from the draft sequences of the *japonica* cultivar Nipponbare and *indica* cultivar 93–11 [3, 4]. The SNP pools were mainly limited to two varieties. Another SNP discovery set from the OryzaSNP project has identified approximately 160,000 high-quality SNPs and has provided more insights by detecting informative SNPs across 20 diverse rice varieties [5]. Next-generation sequencing at 19× coverage across 517 rice varieties has identified over 3.6 million SNPs, of which 167,000 SNPs were located within coding regions [6]. Recently, rice re-sequencing projects have been conducted for various germplasm [7–9].

The decreasing cost, along with the rapid advancement of next-generation sequencing technology and related bioinformatics resources, has facilitated the large-scale discovery of SNPs in various plant species. Genotyping-by-sequencing (GBS) is a next-generation-sequencing based method that takes advantage of reduced representation to enable high-throughput genotyping of a large number of SNP markers. GBS has been applied in SNP genotyping for quantitative trait loci (QTL) mapping and gene identification in rice [10–12]. The TASSEL-GBS pipeline successfully fulfills the following key design criteria: (1) Ability to run on the modest computing resources, including desktop or laptop machines with only 8–16 GB of RAM; (2) Scalability from small to extremely large studies, in which hundreds of thousands or even millions of SNPs can be scored; (3) Applicability in a fast-breeding context, in which rapid genotyping is required due to the high frequency of tissue collection [13].

A total of 12 parental lines were selected for GBS because of their specific characteristics, including grain quality in rice breeding. Guichao2 and IR24 are two varieties with *indica* rice characters such as seed type and disease resistance [14]. Guichao2 was of interest because of its wide range of adaptability, high yield potential, and could be planted as an early, or middle, or late variety. However, this particular variety was later excluded because of bad grain quality. Koshihikari and Asominori are varieties from Japan, and USSR5 is a variety from Russia that shows typical *japonica* rice characters such as good quality, but is susceptible to rice strip virus disease (RSVD) [15]. Sasanishiki is a high-yielding *indica* cultivar [16]. Habataki has very high yield potential and a short, stiff stem, but is susceptible to cold during booting and early growth [17]. Kasalath harbors resistance genes against standard differential blast isolates from the Philippines and Japan [18]. Nanjing35 has superior grain yield, but poor appearance. N22 possesses QTL/genes related to dormancy [19]. RILs and NILs derived from these parental varieties are currently under investigation for genes controlling agronomic traits; therefore the SNP database will be very useful for gene mapping and isolation by using high-density SNP markers. The number of recombination events and the marker density of parental varieties determine the resolution of gene mapping. By using the cost-effective GBS approach, we have detected SNPs among the parents of 12 rice recombination line populations.

The P450s in biosynthetic pathways play critical roles in the synthesis of lignins, pigments, defense compounds, fatty acids, hormones, and signaling molecules [20]. CYP704 shows higher homology with CYP86, CYP94, CYP96 compared to the other P450s, and these are all non A-type P450s belonging to CYP86 [21]. CYP704B2 is involved in anther cutin biosynthesis and pollen exine formation in rice [22]. CYP704A3 is a member of the CYP family, and most of its family members are located in the ER [20], including CYP78A13, which influences grain size and yield in rice [23]. We herein report SNPs for the parental varieties of mapping populations and a miRNA regulated gene, *CYP704A3*, underlying rice seed size.

## Results

### Sequencing and variation calling

**Fig. 1:**
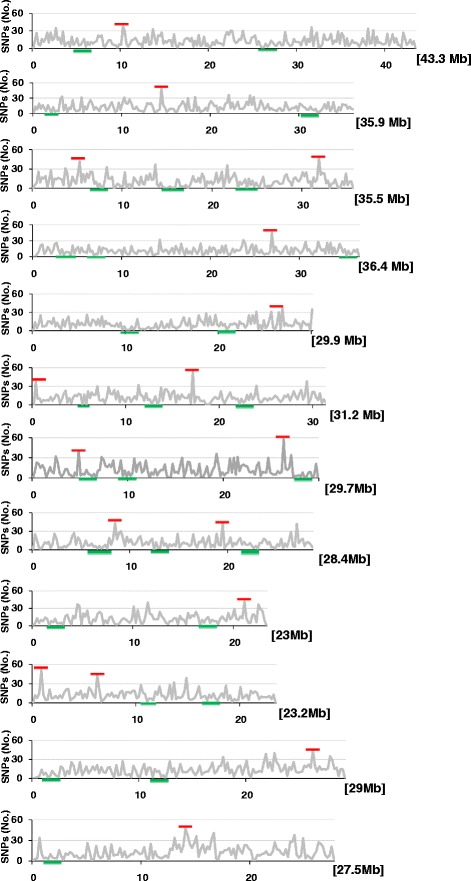
Distribution of SNPs in 12 chromosomes of different rice varieties. The x-axis represents the physical distance along each chromosome, which is split into 200-kb windows. The total size of each chromosome is shown in *brackets*. The y-axis indicates the number of SNPs. The regions with relatively high and low density are labeled in *red and green bars*, respectively

We identified a total of 22,682 polymorphic SNPs in 12 parental varieties and Nipponbare in relation to the reference genome (Additional file 1: Table S1). To explore the genomic distribution of the patterns of DNA polymorphisms between the *indica* and *japonica* subspecies, the SNP count based on our sample was plotted along each chromosome (Fig. 1). SNP count (Fig. 1, solid line) was defined as the number of SNPs in a 200-kb interval. Non-random patterns of SNP distribution were observed, with highly different SNP frequencies detected on all chromosomes. These data support previous findings that polymorphisms in the rice genome (from the *indica-japonica* perspective) are non-randomly distributed [24]. Relatively low SNP polymorphisms were observed in the regions highlighted in green bars (Fig. 1), which could be due to stringent recombination restrictions, or lack of restriction enzyme sites or reads in these regions. More markers need to be developed in these low-density SNP regions for QTL/gene mapping.

We aimed to detect SNPs in the difference rice varieties by using the TASSEL GBS pipeline. Furthermore, we validated 12 SNP genotypes (Additional file 2: Table S2) from 12 chromosomes, respectively, by using Sanger sequencing on the ABI3730xl DNA sequencher (ABI, CA, USA) with the BigDye V3.0 kit. The SNP database with a low rate of missing data will be useful for QTL detection in the populations derived from the 12 varieties.

**Fig. 2:**
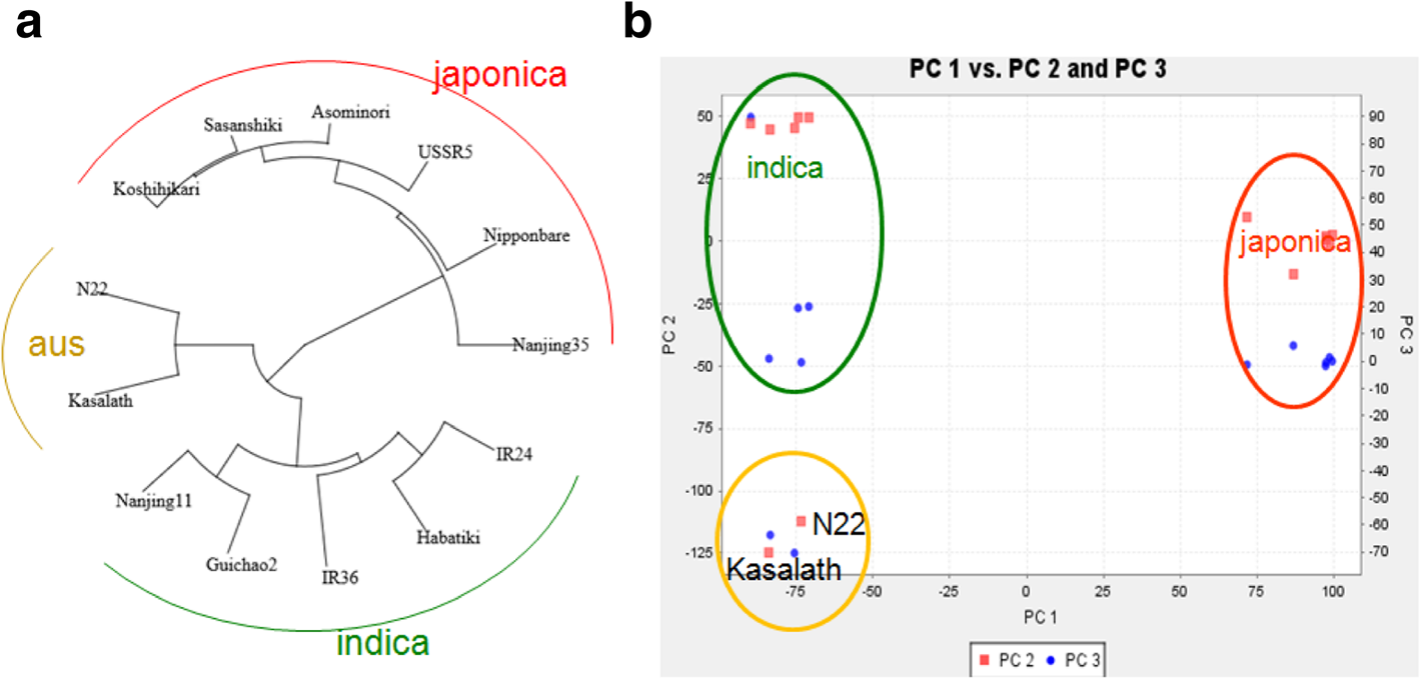
Genetic structure and population differentiation in 13 rice parental varieties. **a** Unweighted pair group method with arithmetic mean (UPGMA) dendrogram based on 22,682 SNPs. **b** Principal component analysis for the entire set of RILs (*n* = 306)

The phylogenetic tree produced using the 22,682 SNPs revealed three distinct groups, with japonicas clustered into one group, and the other two groups together with *aus* and *indica* types (Fig. 2a). Three distinct groups were identified by principal component analysis with well-separated lines, corresponding to *indica*, *aus*, and *japonica* rice species (Fig. 2b). Consistent with the phenotypical classification, the varieties from China, Guichao2, Nanjing11 and Nanjing35 were grouped into the *indica* type, together with IR36 and IR24 from IRRI; the varieties from Japan, Sasanishiki, Koshihikari, Habataki, and Asominori were grouped into *japonica*, together with USSR5 from Russia; the varieties N22 and Kasalath from India were grouped into *aus*. Based on this classification, more SNP polymorphisms could be expected between the parental lines with distinct relationships. Thus, germplasm classification of the 12 rice varieties was properly conducted and the results were informative for further RIL or NIL construction.

We checked 12 SNP genotypes showing the same genotypes within *indica* and *japonica* subgroups by using Sanger sequencing on the ABI3730xl DNA sequencher (ABI, CA, USA) using the BigDye V3.0 kit. Sequences generated by each primer pair were aligned using Sequencher and SNPs were validated by visual inspection.

### SNP annotations and large-effect SNPs

The SNPs were annotated and classified based on their location in intergenic regions, introns, 5′-UTR, 3′-UTR or exon (63.1, 8.5, 4.1, 4.8, and 19.4 %, respectively) (Table 1). We analyzed the SNPs with potentially large effects on gene expression and protein alterations.

**Table 1:** Summary of SNPs in the 12 varieties

	All	Intergene	Intron	5′-UTR	3′-UTR	Exon
chr1	2703	1545	228	120	217	690
chr2	2205	1367	175	122	89	518
chr3	2099	1281	212	79	100	499
chr4	2033	1239	211	107	92	453
chr5	1626	1010	154	67	81	354
chr6	1927	1232	128	76	111	406
chr7	1746	1092	148	60	67	408
chr8	1613	1057	148	83	49	292
chr9	1463	986	133	70	38	254
chr10	1471	994	131	35	72	287
chr11	1972	1213	148	63	119	490
chr12	1824	1303	117	42	65	327
All	22682	14319	1933	924	1100	4978

The mutant of the cytochrome P450 gene *CYP724B1* accumulates large amounts of mRNA; furthermore, its seed is shorter than that of the wide-type [25]. In the present study, we detected an A/T polymorphism between Asominori and IR24 that was associated with short- and long-grain phenotypes respectively. The SNP in the fourth exon (chr4:28,894,497) is predicted to result in the amino acid alteration of Aspartic acid to Valine. These findings suggest that *CYP704A3* might be associated with grain length. Thus, we decided to further investigate a protein-altering A/T polymorphism located in the exon of the cytochrome P450 gene, *CYP704A3* (LOC_Os04g48460).

**Fig. 3:**
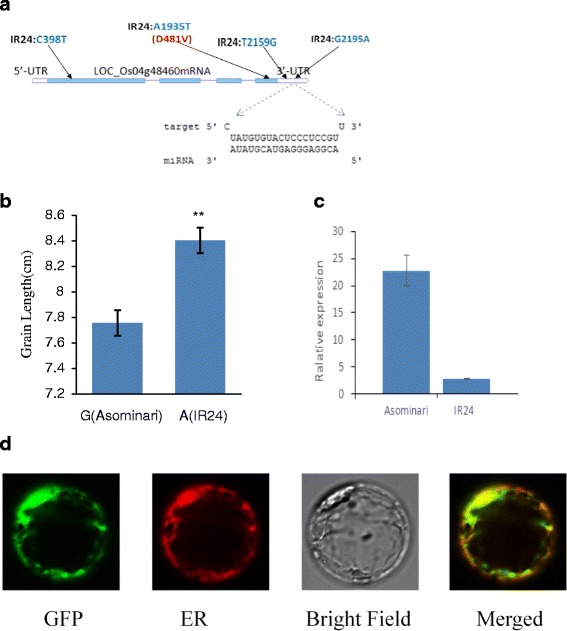
*osa-miRf10422-akr* precursors and its putative target gene. **a** *CYP704A3* is a putative target of *osa-miRf10422-akr. CYP704A3* structure and mutation sites are labeled as SNPs (*blue*) and the changed amino acid residues (*red*). **b** Significant differences in grain length between SNP genotypes of the *CYP704A3* gene. **indicates a significance at *P* < 0.01. **c** qRT-PCR analysis shows the expression of *CYP704A3* in maturing seeds. Asominori and IR24 were analyzed in terms of the expression of the *CYP704A3* transcript. Seeds with longer lengths have lower levels of relative expression, similar to the other members of subfamily CYP450 such as *CYP724B1*, of which its mRNA accumulates at higher levels in *CYP724B1* mutants with shorter seed compared to that in the wide-type. **d** Subcellular localization of the CYP704A3Protein. GFP signals of the CYP704A3-GFP fusion proteins localized in the endoplasmic reticulum of rice protoplasts

We sequenced this gene that exhibited a total of four SNPs, with two present in the exon and two within the 3′-UTR. A G/A SNP (chr4:28,894,757) in the 3′-UTR was detected in the binding region between *CYP704A3* and its miRNA gene, *osa-miRf10422-akr* (Fig. 3a).

We also observed the effects of *CYP704A3* on grain length in a population consisting 184 rice landraces by developing a SNP marker for G/A genotyping the *CYP704A3* gene. The average seed length of plants with the IR24 allele (8.40 ± 0.1 mm) was longer than that harboring the Asominori allele (7.76 ± 0.09 mm) (*P* < 0.01, Fig. 3b). Because of the positive seed length/SNP correlation and the critical position of SNP in the binding region, we deduced that the SNP mutation was crucial to grain size.

Seeds with *CYP704A3* of IR24 allele genotype were longer than that of Asominori allele (Fig. 3b). We further compared the expression levels of *CYP704A3* in maturing seeds of IR24 and Asominori with long and short seeds respectively. The seeds of IR24 with longer size had relative lower expression of *CYP704A3* (Fig. 3d).

To verify the subcellular location of CYP704A3, we constructed a CYP704A3-GFP fusion expression vector, and then transformed the recombinant expression vector into rice protoplasts. Confocal microscopy showed that the green fluorescent signals of CYP704A3-GFP co-localized with the autofluorescence signals of the ER (Fig. 3f).

**Fig. 4:**
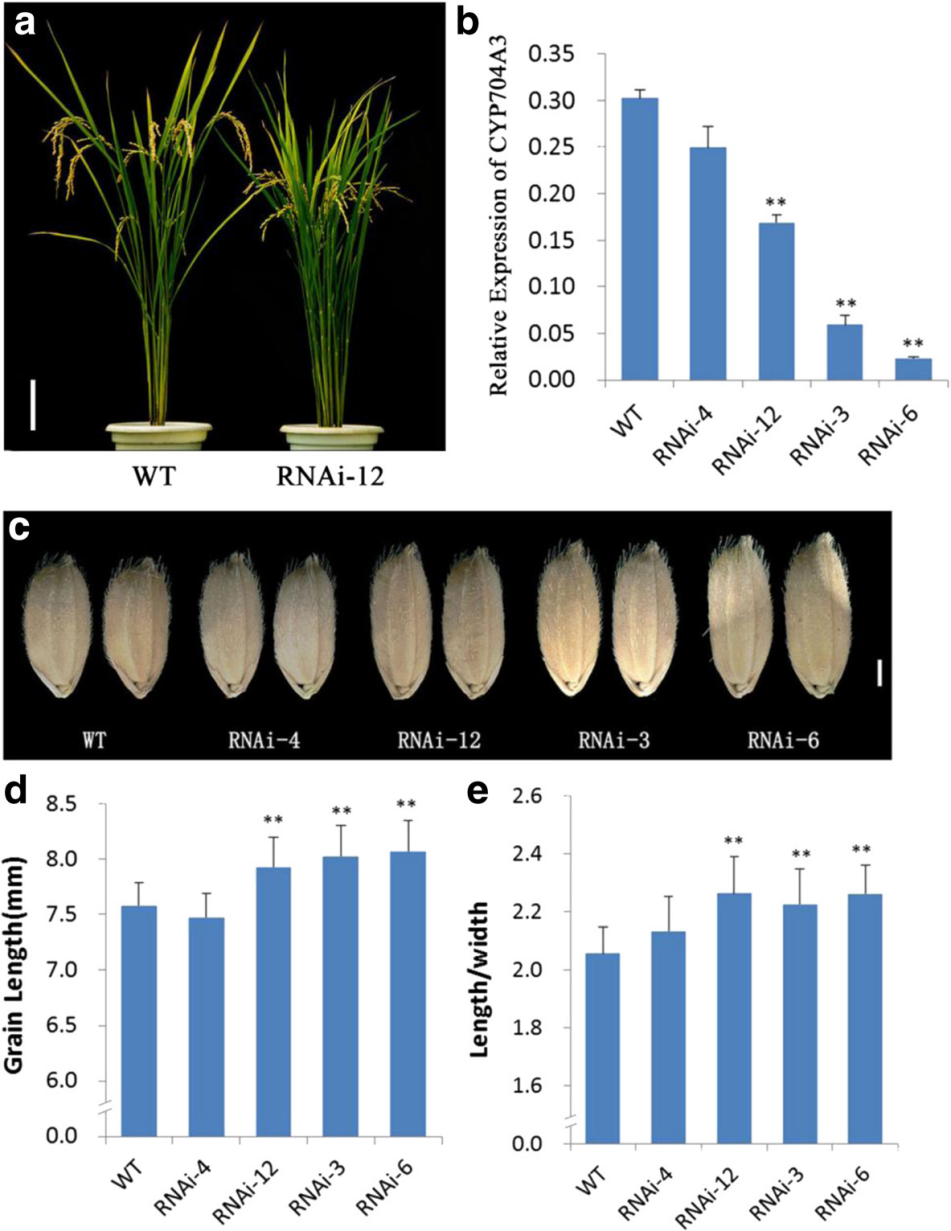
CYP704A3-RNAi transgenic plantsproduced longer grains. **a** The morphology of wild-type and RNAi-12 transgenic plants. Scale bar: 10 mm. **b** Relative expression levels of *CYP704A3* mRNA of the wild-type RNAi-4 (as a transgenic negative control) and T1*CYP704A3*-RNAi transgenic plants which were determined by qRT-PCR. **c** Grains of the wild-type, RNAi-4 (as a transgenic negative control), RNAi-12, RNAi-3 and RNAi-6. Scale bar: 1 mm. **d** Grain length (*n* = 100). **e** Grain length to width ratio (*n* = 100)

To further confirm the function of *CYP704A3*, CYP704A3-RNAi transgenic rice plants were generated. Several independently transformed plants showed a reduction in the level of expression of the *CYP704A3* gene (Fig. 4a). Three of these were selected to represent the relative expression of *CYP704A3* in relation to the transgenic negative control RNAi-4 (Fig. 4b). The reduced expression of CYP704A3 caused various degrees of elongation in grain length (Fig. 4c). Significant difference (*P* ≤ 0.01) in grain length (Fig. 4d) and length/width ratio (*P* ≤ 0.01) (Fig. 4d) among the wild-type, RNAi-4, and *OsCYP704A3*-RNAi transgenic plants were observed. These results further indicated that *CYP704A3* negatively regulates grain length in rice.

### Interaction between *osa-miRf10422-akr* and the *CYP704A3* target gene

**Fig. 5:**
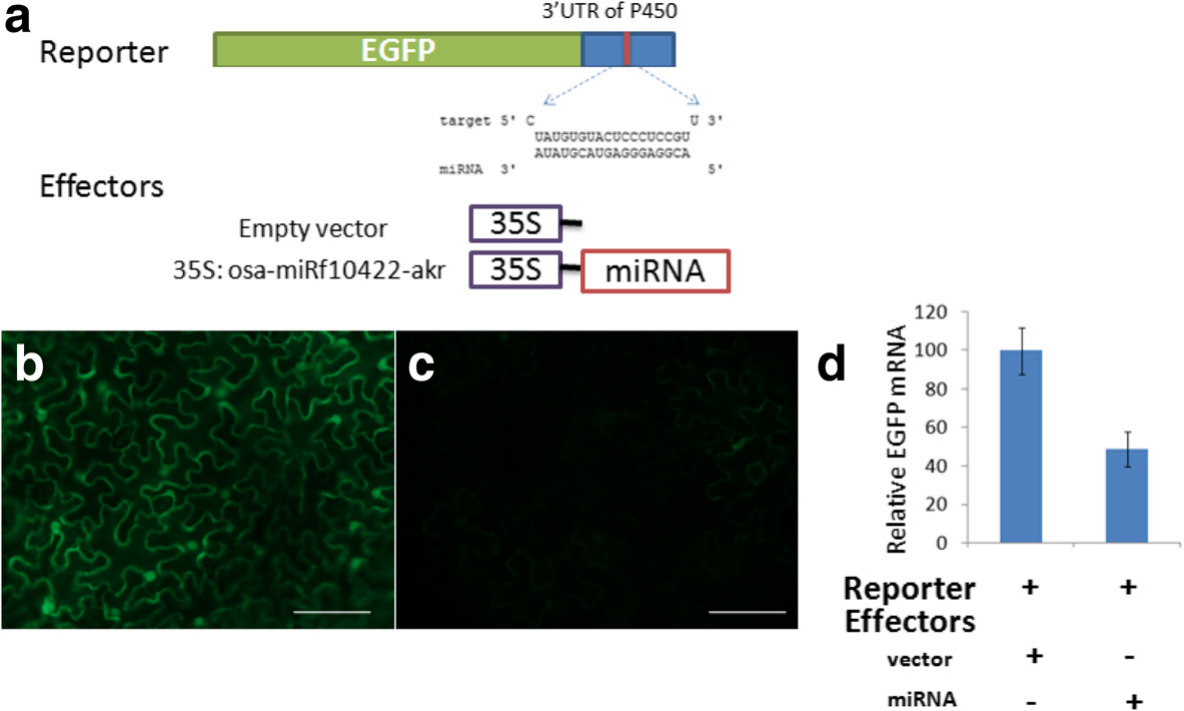
The effects of *osa-miRf10422-akr*expression on the accumulation of the *CYP704A3* gene. The schematic representation of the reporters and the effectors used in this assay is shown in (**a**). GFP fluorescence images of the co-expression of osa-*miRf10422-akr* with the reporter gene EGFP, which was fused with the empty vector control (**b**) and miRNA target region in the 3′-UTR of the CYP704A3 gene (**c**). Fluorescence imaging analysis of the agroinfiltrated leaves at 2 dpi under UV illumination. Quantitation of *EGFP* mRNA as averaged from three leaves from each infiltration treatment (**d**)

To validate that *CYP704A3* is regulated by *osa-miRf10422-akr*, a schematic representation of the reporters and the effectors (Fig. 5a) was used in this assay. To examine the ability of plant expression vectors to produce *osa-miRf10422-akr* miRNAs in vivo, an *Agrobacterium* strain harboring pCAMBIA1300-35S:osa-miRf10422-akr or the control pCAMBIA 1300 vector (*35S*) was infiltrated into *N. benthamiana* leaves, together with the reporter gene *EGFP* which was fused with the 3′-UTR of the rice *CYP704A3* gene, which contained the putative miRNA target. When the effecter recognizes the miRNA target in the reporter construct, the mRNA level of *EGFP* and the fluorescence of EGFP are downregulated. The fluorescence of the agroinfiltrated leaves was taken at 2 dpi under UV illumination. Fluorescence imaging showed that EGFP and *osa-miRf10422-akr* were co-expressed (Fig. 5b) and together with miRNA target region in the 3′-UTR of the *CYP704A3* gene (Fig. 5c). Total RNA was extracted at 3 dpi, and quantitative *EGFP* mRNA analysis using the average measurements of three leaves utilized in each infiltration treatment (Fig. 5d). As expected, *osa-miRf10422-akr* expression affected the *CYP704A3* gene expression both at the transcriptional and protein levels. These findings therefore indicate that *osa-miRf10422-akr* participates in seed size determination by directly regulating *CYP704A3*.

### The *CYP704A3* target gene underwent selection for seed size improvement

**Fig. 6:**
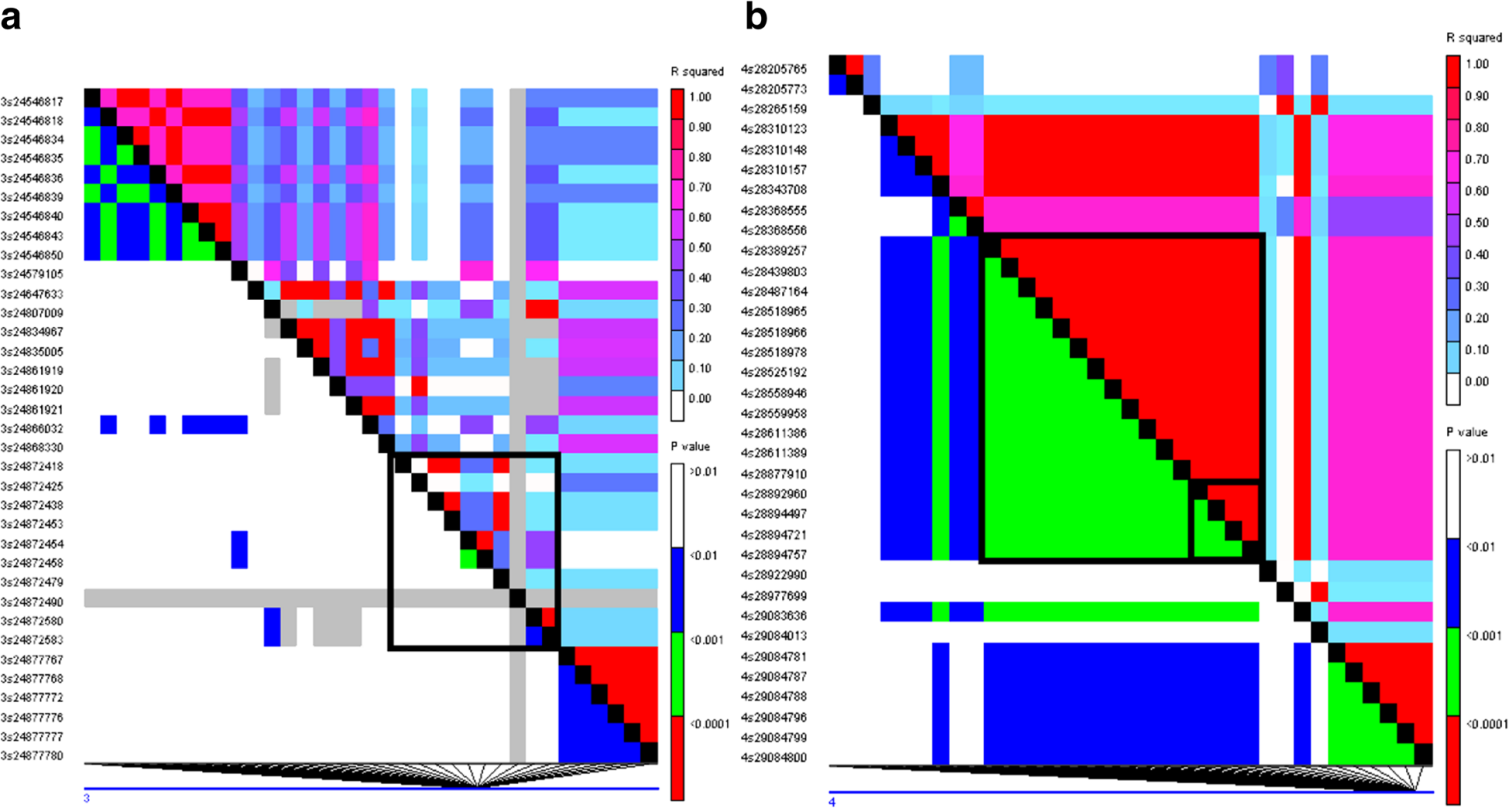
Patterns of LD blocks in two genomic regions of *osa-miRf10422-akr* precursors and its putative target gene, LOC_Os04g48460. **a** No LD blocks in the *osa-miRf10422-akr* region (~24,872Kb) on chromosome 3 were detected. **b** LD block in the *big black block* encompassing the LOC_Os04g48460 region (~28,892 kb in *small black block*) on chromosome 4. *Red and white spots* indicate strong (*r* ^*2*^ = 1) and weak (*r* ^*2*^ = 0) LD, respectively

To test whether the *CYP704A3* target gene and the *osa-miRf10422-akr* had undergone selection within the parental varieties, we calculated the linkage disequilibrium (LD) of the two genes and its flanking regions on both sides using our GBS data. LD analysis of the two genomic regions, namely, *osa-miRf10422-akr* and its putative target gene, *CYP704A3*, of these parental varieties, revealed different patterns of LD blocks (Fig. 6). The *r*^*2*^-values for the miRNA *osa-miRf10422-akr* did not show any increase in these parental varieties that are commonly used in rice breeding. No LD blocks were detected in the *osa-miRf10422-akr* region (~24,872 kb) on chromosome 3 (Fig. 6a). In contrast, the *r*^*2*^-values of the *CYP704A3* gene increased in these parental varieties. A strong LD block was detected in the target gene LOC_Os04g48460 on chromosome 4 (Fig. 6b). The detection of a strong LD block in *CYP704A3* compared to that in the miRNA gene indicated that the *CYP704A3* target gene may have undergone selection within these parental rice varieties, although additional investigations should be performed.

## Discussion and conclusions

De novo genome sequencing or re-sequencing generates the gigabytes of data that need to be analyzed at a very large scale. On the other hand, smaller subsets of SNP data may be utilized for trait-marker analysis and genomic selection for breeding selection. By simplifying genomic data by using GBS, linkage and linkage disequilibrium may be cost-effectively analyzed, and deep candidate gene re-sequencing may be conducted during targeted SNP detection [26]. The genome can be significantly simplified by restriction enzyme digestion. The SNPs can be detected at restriction-associated sites. A higher number of short-read alignments at regions of interest may help in more precisely resolving the real allele frequency of mutant alleles in bulked DNA [26]. Therefore, GBS may be employed for further gene fine-mapping and allele analysis.

SNP markers of *CYP704A3* gene were developed for genotyping a population consisting of 184 landraces, and significant mean differences in the *t*-test suggested that the gene may play roles in grain development. In rice, at least five proteins have been identified through quantitative genetic studies of seed size. Except for the identified novel positive regulator, which is the putative serine carboxypeptidase encoded by GS5 [27], four other proteins are required to limit final seed size and weight [28–31]. In the present study, we observed the upregulation of the *CYP704A3* gene in the short seed variety Asominori, compared to the long seed variety IR24. This result was in agreement with previous findings on the P450 gene *CYP724B1* [25]. *CYP724B1* mRNA accumulated at higher levels in d11 mutants with shorter seeds compared to the wild-type plants [25].

In addition to the cytochrome P450 gene, *CYP724B1*, in rice that controls seed growth, a gene encoding the ortholog of *KLUH*, *SlKLUH*, a P450 enzyme of the CYP78A subfamily in tomato (*Solanumly copersicum*), was reported to affect fruit mass and size [32]. Here, we present evidences indicating that another rice P450 gene, *CYP704A3*, was associated with seed size. Interestingly, *CYP704A3* was found and validated to be a target gene of *osa-miRf10422-akr*. The mechanisms on whether and how the interaction between *osa-miRf10422-akr* and *CYP704A3* controls seed length require further investigation.

In conclusion, we detected a total of 22,682 DNA polymorphisms by high-throughput GBS of mapped reads by assembling these using the reference genomes of *japonica* Nipponbare. Detection of genome-wide DNA polymorphisms by high-throughput GBS enabled us to identify sequence diversity derived from rice differentiation and genomic locations that were related to traits of agronomic importance. We identified polymorphic SNPs in the rice cytochrome P450 gene, *CYP704A3*, which was targeted by a miRNA gene, *osa-miRf10422-akr*, and associated with seed size.

## Methods

### Plant materials

A total of 184 rice accessions were used as study material. The seeds of all accessions were collected, stored and supplied by the State Key Laboratory of Crop Genetics and Germplasm Enhancement of Nanjing Agricultural University, Jiangsu, China. The 184 landraces are collected from eight geographic regions. East China had the most entries, accounting for 27.7 % of the study material, followed by Southwest China (21.2 %), South China (14.7 %), North China (7.6 %), Middle China (8.2 %), Northeast China (5.4 %), Northwest (4.4 %), and Southeast Asia (10.9 %). This study population, including the 12 landraces, was used in the association analysis of seed size. In the past decades, these accessions have been widely used as parents in plant breeding. The 184 accessions were planted from May to November in 2013 and 2014 at the Tuqiao Experimental Farm of Nanjing Agricultural University. For the field experiments, the accessions were grown in a randomized complete block design using two replicates. The space was 20 cm between rows and 17 cm between individuals, with standard agronomic management.

### Sample preparation and sequencing

Genomic DNA was extracted from the 12 rice accessions, including varieties originating from China (Guichao2, Nanjing11, and Nanjing35), Japan (Sasanishiki, Koshihikari, Habataki, and Asominori), India (N22 and Kasalath), and IRRI (IR36 and IR24), and Russia (USSR5). DNA was extracted from leaf tissues using the DNeasy Plant Mini Kit (Qiagen, Germany).

### Sequencing library preparation and sequencing

RAD sequencing is one of several strategies recently developed to improve short-read sequencing by reducing their complexity [33]. RAD sequencing reduces genome complexity by resequencing only the stretches of DNA adjacent to recognition sites of a chosen restriction endonuclease and has been proven to be a powerful tool for genetic analysis [34]. The RAD library of the 12 varieties was prepared for single end-sequencing according to Baird et al. [23] with some modifications. Briefly, barcodes were 6-bp long, being at least two mutational steps separated from each other. A total of 2 μg genomic DNA from each inbred was digested for 1 h at 37 °C in a 50-μl reaction with 50 U of *EcoR*I (New England Biolabs). The RAD library was sequenced on an Ion Torrent PGM and Illumina Hiseq2500. The raw reads that were of high quality were used for the analysis of genetic variations in the 12 accessions.

### Mapping of reads

A large number of reads were assembled based on the genomic sequences of the *japonica* rice cultivars Nipponbare using TMAP3.6. SNPs were detected by comparison alignment using the Nipponbare sequences as reference. Parameters were set as default to classify whether mismatches were sequencing errors or genomic variations.

### SNP detection and analysis

Reads were separated by barcode and trimmed at the 3′ ends. The RAD tags at the RAD clusters were screened for SNPs and InDels using *Oryza sativa* L. cv. Nipponbare (http://rapdb.dna.affrc.go.jp/download/irgsp1.html) as reference. SNPs of each sample were collected using the TASSEL pipeline [27]. Filtering and imputation procedures were performed to call the first 64-bp of the high quality reads with default parameters in the pipeline. A phylogenetic tree was produced using the 22,682 SNPs to show the relationships among the 13 landraces. The SNP cladogram-tree dataset was generated using the neighbor-joining method as provided in TASSEL [35]. Three distinct groups were identified by principal component analysis using TASSEL [35]. LD of the two genes and its flanking regions on both sides was calculated using our GBS data using TASSEL [35].

A dCAPs marker was developed for the SNP in the binding region between *CYP704A3* and its miRNA gene, *osa-miRf10422-akr*. The average seed length of plants with T identical to IR24 was compared to the A that was identical to Asominori using the *t*-test, using a significance level of *P* < 0.01.

### Polymorphism verification and dCAPs marker genotyping

Twelve polymorphic RAD clusters that were located in the 12 chromosomes were randomly selected. Primers were designed to flank the entire RAD cluster. The target sequence was amplified in all 12 varieties. The PCR conditions were as follows: 94 °C for 3 min; followed 35 cycles of 94 °C for 30 s, 55 °C for 30 s, 72 °C for 30 s; 72 °C for 5 min, and then held at 4 °C. Sanger sequencing of PCR products was conducted using an ABI3730xl DNA sequencer, following standard protocols. Primers for *CYP704A3* were as follows: Forward 5′-CAAGGGCGGCGCTGGTCTATT-3′ and Reverse 5′-ATTTTCCTTTGGTTATGTTTTGTA-3′.

### Real-time PCR and subcellular localization of the CYP704A3 protein

Total RNA was isolated from maturing seeds using a plant RNA purification reagent (Invitrogen). Synthesis of cDNA and real-time PCR were performed as described elsewhere [36]. The rice Actin gene was selected as endogenous reference. PCR specificity was examined by 3 % agarose gel electrophoresis using 5 μL of each reaction to check the right product length and to make sure that no primer dimers or non-specific amplicons were generated. The primers for real-time PCR were as follows: Forward 5′-GTCGCCTTGTCGCTGCTGCTAC-3′ and Reverse 5′-CGGGCGGATACCTGCGTTTCT-3′ for the *CYP704A3* gene.

For the subcellular localization of the CYP704A3 protein in rice protoplasts, the coding sequence of the *CYP704A3* gene was amplified and inserted into the *Bgl*II/*Not*I sites of the PA7 vector to form a translational fusion with the C-terminus of the GFP. The transient expression constructs were transformed into rice protoplasts as described elsewhere [37]. The fluorescence of GFP was observed using a confocal laser scanning microscope (Leica TCS SP5). The primers used in subcellular localization assays were as follows: Forward: 5′-ATGGACGAGCTGTACAGATCTATGGAGTCGCCGCT-3′ and Reverse: 5′-GAACTGCAGCCGGGCGGCCGCTCACCGGGCCAATG-3′.

### Rice transformation

To validate the function of *CYP704A3*, a *CYP704A3*-RNAi vector was constructed and introduced to wild-type plants. To construct the RNAi vector, a 316-bp fragment within the CDS was amplified using the forward primer, 5′-GGGGTACCTCCGGCGGCGAAGG-3′ and reverse primer, 5′-CGAGCTCTTGCTCTCTGCTCATCTG-3′ with the *Kpn*I and *Sac*I enzyme digestion sites, and the reverse sequence was amplified using the forward primer, 5′- GGTACGTATCCGGCGGCGAAGG-3′ and reverse primer, 5′-AACTGCAGTTGCTCTCTGCTCATCTG-3′ with the *SnaB*I and *Pst*I enzyme digestion sites.

The rice plants examined under natural field conditions were grown in normal rice growing seasons at the Experimental Station of Nanjing Agricultural University, Nanjing, China. Seeds were planted in a seedbed in mid-May and transplanted to the field in mid-June 2015. Field management, including irrigation, fertilizer application, and pest control, was essentially performed using standard agricultural practices. Harvested paddy rice was air-dried and stored at room temperature prior to testing. Fully filled grains from each plant were randomly chosen (*n* = 100) for grain size evaluation.

### Quantitative EGFP fluorescence and miRNA analysis

GFP fluorescence imaging of the coexpression of *osa-miRf10422-akr* with the reporter gene EGFP fused with the empty vector control and the miRNA target region in 3′-UTR of the *CYP704A3* gene was performed. The fluorescence of the agroinfiltrated leaves was examined at 2 dpi under UV illumination. Quantitative EGFP mRNA of three leaves from each infiltration treatment was also analyzed.

### Availability of data and materials

The data sets supporting the results of this article are included within the article and its additional files. All the sequencing data produced in this study have been deposited in NCBI Short Read Archive (http://www.ncbi.nlm.nih.gov/sra/) and can be accessed under the SRA accession numbers: SRR3307074, SRR3307908, SRR3308144, SRR3308415, SRR3308416, SRR3308417, SRR3308419, SRR3308421, SRR3308737, SRR3310108, SRR3310109, SRR3310110, SRR3310115, SRR3310157, SRR3310158. SRR3310111, SRR3310156, and SRR3308736.

## Additional files

Additional file 1: Table S1. 22,682 polymorphic SNPs among the 12 parental varieties and Nipponbare as the reference genome. (XLSX 1756 kb) Additional file 2: Table S2. Primers for 12 SNP genotypes from the 12 chromosomes respectively, using Sanger sequencing on the ABI3730xl DNA sequencher (ABI, CA, USA). (PDF 95 kb)

## Abbreviations

3′-UTR: 3′-untranslated region
EGFP: enhanced green fluorescent protein
ER: endoplasmic reticulum
GBS: genotyping-by-sequencing
LD: linkage disequilibrium
QTL: quantitative trait loci
RAD: restriction-site-associated DNA
RIL: recombinant inbred line
SNP: single-nucleotide polymorphism
