# Erratum to: SNP-based analysis of genetic diversity reveals important alleles associated with seed size in rice

**DOI:** 10.1186/s12870-016-0801-9

**Published:** 2016-06-07

**Authors:** Weijie Tang, Tingting Wu, Jian Ye, Juan Sun, Yue Jiang, Jun Yu, Jianpeng Tang, Gaoming Chen, Chunming Wang, Jianmin Wan

**Affiliations:** State Key Laboratory of Crop Genetics and Germplasm Enhancement, Nanjing Agricultural University, 210095 Nanjing, China; State Key Laboratory of Plant Genomics, Institute of Microbiology, Chinese Academy of Sciences, 100101 Beijing, China; Jiangsu Collaborative Innovation Center for Modern Crop Production, Nanjing, China; National Key Facility for Crop Gene Resources and Genetic Improvement, Institute of Crop Science, Chinese Academy of Agricultural Sciences, Beijing, 100081 China

## Erratum

Unfortunately, the original version of this article [1] contained two errors within the text. On page 2 within the Background section the sentence: “Sasanishiki is a high-yielding indica cultivar” should have read: “Sasanishiki is an average-yielding japonica cultivar”.

Also at the end of page 2 in the Results section the sentence: “Consistent with the phenotypical classification, the varieties from China, Guichao2, Nanjing11 and Nanjing35 were grouped into the *indica* type, together with IR36 and IR24 from IRRI; the varieties from Japan, Sasanishiki, Koshihikari, Habataki, and Asominori were grouped into *japonica*, together with USSR5 from Russia; the varieties N22 and Kasalath from India were grouped into *aus*.”should have read: “Consistent with the phenotype classification, the varieties from China, Guichao2 and Nanjing11 were grouped into the *indica* type, together with IR36 and IR24 from IRRI; the varieties from Japan, Sasanishiki, Koshihikari, and Asominori were grouped into *japonica*, together with USSR5 from Russia; the varieties N22 and Kasalath from India were grouped into *aus*.”

## References

[1] Weijie T, Tingting W, Jian Y, Juan S, Yue J, Jun Y, Jianpeng T, Gaoming C, Chunming W, Jianmin W (2016). SNP-based analysis of genetic diversity reveals important alleles associated with seed size in rice. BMC Plant Biol.

