# Supplementary material for: SNP-based analysis of genetic diversity reveals important alleles associated with seed size in rice
Source: BMC Plant Biol. 2016 Apr 19;16:93. doi: 10.1186/s12870-016-0779-3 (PMC4837510; doi:10.1186/s12870-016-0779-3)
Supplement: Additional file 2: Table S2. — Primers for 12 SNP genotypes from the 12 chromosomes respectively, using Sanger sequencing on the ABI3730xl DNA sequencher (ABI, CA, USA). (PDF 95 kb) [file 12870_2016_779_MOESM2_ESM.pdf]

Supplementary Table 2 Primers for twelve SNP genotypes from the twelve chromosomes respectively, using Sanger sequencing on the ABI3730xl DNA sequencher (ABI, CA, USA)

Alleles	Chromosome	Position	Primer	
C/T	1	4328	F	CAGGCAGAGAATTTTAGGGAGATA
			R	GACAATGAATTTTAGGGCTTTACG
T/A	2	27572325	F	ATGAAATCCGCAACTATGTGGT
			R	AACGTGCCTATGCCTAATGAAGAT
C/T	3	33988653	F	AGTCACGATTGCCTCACCACCAT
			R	GGCCTCCCTAGCATATTTGACTCC
C/T	4	8214531	F	ATGCTTCGCCGTTGCCTTCT
			R	TTTTCGTTAGCGTGGTTCGTTGAT
G/A	5	13992737	F	TTGGCCTAAAACCTGTGAGC
			R	ATCCGTATTGCGACTTGAAAACTT
G/A	6	21031848	F	CGCCTACCCCTACGCCTACG
			R	CTCTCCTCCCGCTTGTGAATGTT
A/G	7	8524028	F	TGGCATCAGCGGTCTTTC
			R	AGGGATTTAACGGCTTGTCA
C/T	8	4647706	F	TCCCGCAGCTAAGCATAAGATT
			R	CGCCGGGGTTTCAGCAGT
C/T	9	3063991	F	TGGAGGCGTCGTAGTCGGTAATGT
			R	GAGGCGACGTCGTGGCTTCTGTTC
C/T	10	7017746	F	CTGTTACCGGCTTTGTTTCTGTG
			R	CCATGACCCGGTTTTTGTAGG
T/C	11	18434679	F	TTGAAAGTGGGAAATGTTGACC
			R	AGAAAGTACGTATGATCGGAAAAA
A/C	12	25828739	F	TAGCCGCCATCCTTCCATTG
			R	ACGGTTTTCAGATATTGTCCAC
